# Case Series: Severe meibomian gland loss in polycystic ovarian syndrome patients on estrogen-progesterone therapy

**DOI:** 10.12688/f1000research.139229.1

**Published:** 2023-09-14

**Authors:** Japmehr Kaur Sandhu, Swati Singh, Sayan Basu

**Affiliations:** 1 Centre for Ocular Regeneration, LV Prasad Eye Institute, Hyderabad, Telangana, 500034, India; 2 Opthalmic Plastic Surgery Services, LV Prasad Eye Institute, Hyderabad, Telangana, India

**Keywords:** Meibomian gland, Dry eye disease, Sex Hormones, Hormone replacement therapy

## Abstract

**Purpose:** To report the ocular surface and meibomian gland changes in polycystic ovarian syndrome (PCOS) women taking hormone supplementation.

**Methods:** Case series.

**Results:** Three women (27 ± 11 years) already diagnosed with PCOS presented with dry eye symptoms (mean OSDI, 37.5) for a mean duration of 13 months and were taking hormonal supplements for a mean duration of 60 ± 11 months. The hormonal supplements included oral estrogen (n=3), oral progesterone (n=3), antiandrogen cyproterone (n=1) and isotretinoin (n=1). Ocular surface evaluation revealed mean NIBUT of 9.9 ± 1.6 seconds and mean TMH of 0.27 ± 0.05 mm, assessed non-invasively using Oculus keratograph 5M (K5M). Meibography (K5M) showed near total loss of all meibomian glands (n=8/12 eyelids) with residual ghost glands in all four eyelids of two patients, and gland shortening alone in one patient. The gland morphology did not change following intense thermal pulsation treatment or cessation of hormonal therapy.

**Conclusions:** Near-total irreversible meibomian gland loss was seen in two young PCOS women taking hormonal supplements. Collaboration between ophthalmologists and gynecologists is advisable for early detection and better understanding of dry eye disease (DED) progression in these patients.

## Introduction

Polycystic ovarian syndrome (PCOS) is one of the commonest endocrine abnormality in women of reproductive age. An association between PCOS and ocular surface alterations has been proposed due to hormonal imbalance and hyperandrogenism seen in PCOS. ^1^^–^^6^ Many components of the ocular surface are influenced by sex hormones, including the lacrimal gland, meibomian glands, and cells of the conjunctiva and cornea. ^7^ In PCOS, increased mucus production, allergy-like symptoms and conjunctival inflammation have been observed. ^4^ Published data has shown increased meibomian gland dysfunction (MGD) in PCOS that had no correlation with testosterone levels. ^1^ The reported changes in meibomian glands in PCOS were average 20% loss in meibomian gland area in the lower eyelid, elevated OSDI score, and reduced tear stability. ^1^^–^^6^ Meibomian gland epithelial cells have been shown to express sex-steroid hormone receptors (especially estrogen, androgen) that affect lipid synthesis within the glands. However, the effects of hormone replacement therapy (HRT, estrogen and progesterone) on meibomian gland function of post-menopausal women are variable, with few reporting improvements, while others reported worsening with HRT. ^8^^,^^9^ To the best of our knowledge, the effects of HRT on the ocular surface of PCOS women have not been reported before. The authors present three such cases where severe meibomian gland loss and dry eye disease (DED) was observed in women with PCOS taking prolonged hormonal supplementation.

## Case series

### Demographics and history

**Figure 1.**
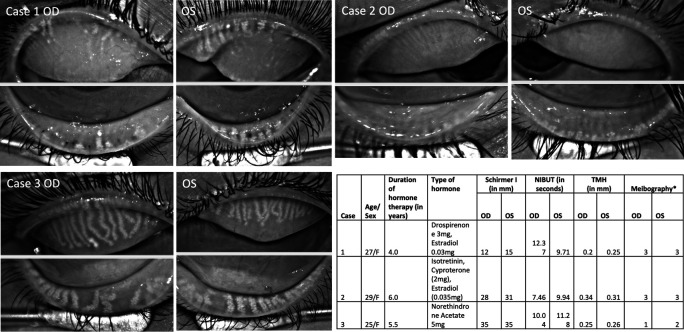
Case 1 Meibography shows severe gland loss with ghost and thin glands in the upper and lower eyelids of both right (OD) and left (OS) eyes. Case 2 with severe meibomian gland loss in the right and left eye. Case 3 has moderate gland loss in both upper and lower eyelids of both eyes. Table summarizes details of three PCOS patients along with tear film details. NIBUT=Non-invasive tear break-up time; TMH=Tear meniscus height; OSS= Ocular staining score; F=Female; * indicates grading system by Arita et al. 2016.

Three women (mean age, 27±2 years) of Indian origin diagnosed with meibomian gland dysfunction who were taking hormone supplements for polycystic ovarian dysfunction were included ( Figure 1). One of the women was a homemaker and the rest two were students. There was no significant family history of autoimmune DED. The chief complaints were dryness/irritation (n=2), pain (n=1), burning sensation (n=2), floaters (n=1) and a ‘pulling sensation’ in both eyes (n=1). All had bilateral ocular involvement with DED symptoms. The mean duration of ocular symptoms was 13 months, and they were using hormone therapy for the mean duration of 60±12 months. Two were using oral estrogen and progesterone prescribed for their polycystic ovarian disease (PCOD) and one was a chronic user of isotretinoin along with oral estrogen and progesterone (Case 2; cyproterone (2 mg) and ethyl estradiol (0.035 mg)) for acne with PCOD.

### Meibography

Meibography showed severe gland loss in both eyes of two patients ( Figure 1; Case 1 and 2) whereas moderate loss in one patient ( Figure 1; Case 3). The changes were equally distributed in the upper and lower eyelids. In 8/12 eyelids, the glands had a ghost-like appearance where only the terminal ductal opening was hyperreflective in few of them. The remaining visible glands were thinned out in these two patients.

## Tear film and ocular surface changes

The mean non-invasive tear break-up time (NIBUT) was 9.9 ± 1.6 seconds. None of the eyes had aqueous deficiency as mean tear meniscus height (TMH) value was 0.27 ± 0.05 mm and mean Schirmer without anesthesia was 24 ± 10 mm ( Figure 1). Meibomian glands were not expressible in two patients and clear meibum in third patient (Case 3). Ocular surface did not show any congestion but had ocular staining score of 1 in all of them. One of the patients had lagophthalmos of 2 mm and inferior corneal scarring (Case 2).

### Treatment

All patients were put on sodium hyaluronate 0.18% eye drops. Case 3 with DED and exposure keratopathy changes was prescribed additional eye drops (containing hydroxypropyl methylcellulose 0.3%, dextran 70 0.1%, glycerin 0.2%). Case 2 underwent lipiflow therapy but showed no improvement in symptoms or meibomian gland morphology following treatment. The patients were referred to gynecologist for their respective disorders and dosing schedule of hormonal supplementation.

## Discussion

The present paper describes the severe meibomian gland loss observed in three PCOS women taking hormonal supplementation. Interesting findings in two of the cases were near total loss of meibomian glands in upper and lower eyelids of both eyes with near normal tear break-up time (9 s). This is in contrary to the reported tear film changes seen in PCOS who are not on any hormonal therapy. Almost complete loss of meibomian glands from all eyelids has been rarely reported on meibography in young PCOS women (mean age, 27 years) whether with or without hormonal supplementation. Other than hormonal imbalance of PCOS, oral estrogen intake that downregulates the cell proliferation within the meibomian gland acini and compete with androgens to act on the glands could be responsible for severe loss. One of the patients with acne and PCOS was taking antiandrogens, thus contributing to the gland loss. However, the morphology of gland loss was similar to the women not taking any antiandrogens. Gynecologists can be sensitized to explore DED symptomatology in PCOS patients where they are planning to administer HRT and during follow-up. A detailed ocular surface examination prior to therapy initiation would be helpful. Also, meibomian gland targeted therapies might not be advisable in women taking hormonal supplementation with almost total gland loss as they do not grow back.

The effects of HRT on tear glands are reported mostly in terms of symptoms without much knowledge about the structural changes. We found significant gland loss in our series. DED observed in menopause is supposed to be secondary to the reduced androgens levels in menopause. ^7^ However, PCOS has hyperandrogenism and theoretically it should not result in meibomian gland loss. In the current series, all patients were taking estrogen supplements. The intake of estrogens affects testosterone levels and reduces sebaceous gland growth in the body and decrease sebocyte differentiation via the premature release of lysosomal enzymes. This might be the possible mechanism of HRT action on the meibomian glands in PCOS. Dry eye symptoms are reported in PCOS women, and they have normal Schirmer values and a negative correlation between the gland dropout and plasma estradiol levels. ^1^ In PCOS, hyperandrogenism is expected (which supports acinar proliferation), however, these patients were taking combined hormone therapy and had meibomian gland loss. In PCOS, contact lens intolerance was reported due to increased mucus production. ^4^ In our series, we did not observe presence of mucus threads in the tear lake, which could be due to counteracting effect of HRT. It was surprising to see normal NIBUT values despite extensive loss of meibomian glands in our series. Future studies comparing goblet cell density with other tear film parameters would need to test the hypothesis of whether increased mucus production stabilizes the tear film in these patients.

The majority of studies have refuted any role of HRT in developing dry eye symptoms but the studied interventions (hormonal supplementation) were given only for a duration of one month. Uncu *et al.* reported that DED symptoms start after 12 months of HRT. ^9^ Hence, it may be postulated that prolonged use of HRT may exacerbate symptoms. In our study, women were on hormone therapy for an average duration of five years. In our series, lacrimal gland function was unaffected in all patients whereas meibomian gland loss was noted in all of them. While two patients (Case 1, 2) were on combined estrogen-progesterone therapy, Case 3 was using only norethindrone acetate (synthetic progesterone) 5 mg daily and had gland shortening compared to gland loss seen in rest of the cases. Oral intake of isotretinoin can also affect sebaceous secretions and cause atrophy of sebaceous glands. ^10^ Rather than gland dropout, isotretinoin use is frequently associated with loss in meibomian gland density and size. Case 2 who was on chronic isotretinoin demonstrated normal Schirmer and NIBUT values but showed extensive meibomian gland dropout in both eyes. The pre-HRT therapy meibography images are not available (though all were asymptomatic earlier), hence the timeline of progression of meibographic changes and their relationship with ovarian disease or therapy could not be studied. Nevertheless, observation of near total gland loss and a detailed ocular surface and dry eye work up are the contributions of this case series.

Meibomian gland dropout represents end-stage acinar atrophy leading to the loss of functional glandular tissue inside the tarsal plates, that cannot be reversed with any gland targeted therapies like Lipiflow. Follow-up in one case (Case 2) demonstrated no improvement after two months of discontinuing hormone therapy. Hence, this permanent and severe dropout requires early detection and further investigation of the relationship between the dose or duration of HRT and gland changes. The use of prolonged hormonal supplementation and antiandrogens in PCOS can be associated with severe gland atrophy as noted in our case series. Hence, a careful ophthalmological check-up might be advisable for these women before and during the hormonal therapy especially when they have DED symptoms. As meibomian gland loss is irreversible, an early timely intervention might help in preventing the gland loss. Future studies can explore the correlation between the dosage/duration of hormonal therapy and progression of meibomian gland changes on meibography.

## Ethics approval

Waived as this was a retrospective study.

## Consent for publication

Written informed consent was obtained from all subjects for the use of their electronic data for publication purpose.

## Authors’ contributions

All authors contributed to the conceptualization, data collection and manuscript preparation (writing and editing).

## Data Availability

No data are associated with this article.
