# Supplementary material for: Severe meibomian gland loss in polycystic ovarian syndrome patients on estrogen-progesterone therapy: A case series
Source: F1000Res. 2024 Jul 11;12:1154. Originally published 2023 Sep 14. [Version 2] doi: 10.12688/f1000research.139229.2 (PMC11211728; doi:10.12688/f1000research.139229.2)
Supplement: Peer review history

# Reviewer report: Reviewer response for version 1

**DOI:** 10.5256/f1000research.152489.r281736

**Published:** 2024-06-27

**Authors:** Swaminathan Sethu

**Affiliations:** 1 GROW Research Laboratory, Narayana Nethralaya Foundation, Bangalore, Karnataka, India

The authors with this case series highlight the relevance of a multidisciplinary team and approach in the management of PCOS/PCOD to ensure quality of life for the patient. Hormonal imbalance affects ocular surface homeostasis, in addition to other organs and tissues. Hence, it would be beneficial for patients with PCOS to be simultaneously managed by both gynaecologist and an ophthalmologist. In addition to the case details, the authors have communicated a very balanced discussion.

 The following amendments would further help in improve readership understanding

1. Rephrasing the following sentence would improve the clarity of the statement made. “The reported changes in meibomian glands in PCOS were average 20% loss in meibomian gland area in the lower eyelid, elevated OSDI score, and reduced tear stability”. Suggestion if appropriate: “The ocular surface changes in PCOS patients include increased OSDI score, reduced tear film stability along with a loss of meibomian glands (~20%)”
2. It will be useful to add a citation to the statement “Meibomian gland epithelial cells have been shown to express sex-steroid hormone receptors (especially estrogen, androgen) that affect lipid synthesis within the glands”.
3. Polycystic ovarian syndrome, polycystic ovarian dysfunction and polycystic ovarian disease has been interchangeably used in this manuscript.  Polycystic ovarian syndrome and polycystic ovarian disease are different clinical entities. In the introduction the authors have mentioned it as PCOS and in the demographics & history section it is stated as PCOD. It will be useful if the authors can clarify this aspect.
4. The abstract includes “cessation of hormonal therapy”, however, the details pertaining to this aspect is missing in the “treatment” section of the document. It is mentioned in the discussion section that only subject "case 2" had undergone discontinuation of hormone therapy.  
5. Similarly, intense thermal pulsation treatment was only done in 1 subject. It would be useful to include the regimen followed and details on how long after the intense thermal pulsation treatment was the improvement assessed.
6. It would be helpful for the readers, if any information can be shared with reference to the reason underlying and choice of subject for (a) Cessation of hormonal therapy of 1 subject, and (b) intense thermal pulsation treatment for another subject.
7. If possible, include clinical information (such as those listed in table insert in Figure 1) following ophthalmic care. It can be useful for the readership.

Are enough details provided of any physical examination and diagnostic tests, treatment given and outcomes?

Yes

Is the background of the cases’ history and progression described in sufficient detail?

Yes

Is sufficient discussion included of the importance of the findings and their relevance to future understanding of disease processes, diagnosis or treatment?

Yes

Is the conclusion balanced and justified on the basis of the findings?

Yes

Reviewer Expertise:

Immunology, Dry Eye Disease, Ocular diseases

I confirm that I have read this submission and believe that I have an appropriate level of expertise to confirm that it is of an acceptable scientific standard.

# Reviewer report: Reviewer response for version 1

**DOI:** 10.5256/f1000research.152489.r206936

**Published:** 2023-12-13

**Authors:** Jaime David Martinez

**Affiliations:** 1 Anne Bates Leach Eye Center, Bascom Palmer Eye Institute, Department of Ophthalmology, University of Miami Miller School of Medicine, Miami, FL, USA

I enjoy reading this paper. It does teach the need for ophthalmologists and gynecologists to raise awareness on the causes of dry eye as they mention once meibomian glands atrophy, they do not regenerate. I'd like you to recommend the following changes.

 Paragraph 1 in the discussion's sentence recommends changing the wording. “Also, meibomian gland targeted therapies might not be advisable in women taking hormonal supplementation with almost total gland loss as they do not grow back.” Change the word grow back to regenerate. “Also, meibomian gland targeted therapies might not be advisable in women taking hormonal supplementation with almost total gland loss as they do not regenerate.”

Are enough details provided of any physical examination and diagnostic tests, treatment given and outcomes?

Yes

Is the background of the cases’ history and progression described in sufficient detail?

Yes

Is sufficient discussion included of the importance of the findings and their relevance to future understanding of disease processes, diagnosis or treatment?

Yes

Is the conclusion balanced and justified on the basis of the findings?

Yes

Reviewer Expertise:

Ophthalmology, Cornea , Ocular surface diseases

I confirm that I have read this submission and believe that I have an appropriate level of expertise to confirm that it is of an acceptable scientific standard.
